# The experiences and needs of older adults receiving voluntary services in Chinese nursing home organizations: a qualitative study

**DOI:** 10.1186/s12913-024-11045-5

**Published:** 2024-04-29

**Authors:** Lin Li, Qin Shen, Junxian Wu

**Affiliations:** School of Nursing, Zhejiang Chinese Medical University, 548 Binwen Road, Binjiang District, Hangzhou, 310053 Zhejiang China

**Keywords:** Nursing home organizations, Voluntary, Experience, Needs, Qualitative research

## Abstract

**Background:**

Older adults living in nursing home organizations are eager to get voluntary help, however, their past experiences with voluntary services are not satisfactory enough. To better carry out voluntary services and improve the effectiveness of services, it is necessary to have a deeper understanding of the experiences and needs of older adults for voluntary services.

**Methods:**

The purposive sampling method was used to select 14 older adults from two nursing home organizations in Hangzhou and conduct semi-structured interviews, Collaizzi’s seven-step method was used to analyze the data.

**Results:**

Older adults in nursing home organizations have both beneficial experiences and unpleasant service experiences in the process of receiving voluntary services; Beneficial experiences include solving problems meeting needs and feeling warmth and care, while unpleasant service experiences include the formality that makes it difficult to benefit truly, lack of organization, regularity, sustainability, and the mismatch between service provision and actual demands. The needs for voluntary services mainly focuses on emotional comfort, Cultural and recreational, and knowledge acquisition.

**Conclusion:**

Older adults in nursing home organizations have varied voluntary experiences, and their voluntary service needs are diversified. Voluntary service needs of older adults should be accurately assessed, and voluntary service activities should be focused upon.

**Supplementary Information:**

The online version contains supplementary material available at 10.1186/s12913-024-11045-5.

## Introduction

As a result of advancements in medical technology and improved sanitation conditions, the average life expectancy of Chinese people has increased significantly from 60 years in 1970 to 77.3 years in 2023. However, this has led to a growing number of older adults in China. According to the seventh population census conducted by the National Bureau of Statistics of China, there are now 260 million people over the age of 60 living in the country [1], The aging population in China is growing, and population balance is becoming a core challenge for the country in the long term. The increasing aging population has posed significant challenges and burdens to the state and society [2], China’s aging population challenges the current security system, requiring significant efforts from the state and society for improvement [3].

There are three main modes of old-age care in China: family old-age care, community old-age care, and institutionalized old-age care. Family old-age care is the most traditional form of old-age care in China, due to the reduction in family size and the formation of the “4-2-1” family model - which consists of four older adults, one couple, and one child - the traditional family model is no longer able to meet the growing demand for older adults care [4]; China’s community old-age care is still in the exploratory stage, facing challenges such as slow construction, insufficient staff, and lack of professional knowledge. As a result, it cannot provide meticulous care services for older adults [5]. Against this background, institutionalized older adult care has gradually become popular, it refers to older adults in social service organizations such as senior citizen apartments, welfare homes, and homes for older adults to spend their later life [6]. The challenges of population aging and the inadequacies of family and community support for older adults have resulted in a growing number of older adults opting to reside in nursing home organizations. This has undoubtedly placed additional burdens and challenges on these nursing home organizations. Due to multiple challenges such as late start, low quality, and lack of professional and technical talents, China’s nursing home organizations are still a long way from meeting the comprehensive needs of older adults in terms of health management, skilled nursing care, rehabilitation training, cultural and recreational services, psychological counseling, and social interaction [7]. To tackle the issue of an aging population in China and ensure that older adults have a high quality of life when choosing nursing home organizations, it is necessary to enhance the quality of older adult care services by engaging social forces, such as volunteer teams [8]. Voluntary services refer to the voluntary, unpaid public service offered by individuals, organizations, and voluntary service organizations to society or other organizations. The forms of voluntary services are diverse and can be either formal, planned, and long-term, or informal, spontaneous, and intermittent [9]. At present, volunteer groups in China’s nursing home organizations are mostly informal and consist of university students, healthcare workers, art workers, social workers, and others. These groups are invited by nursing home organizations or come to these institutions on their initiative to provide services for older adults. These services include a wide range of activities such as haircutting, cultural performances, spiritual comfort, hobby learning (e.g., paper-cutting, flower arranging), organizing festive activities (e.g., making rice dumplings on-site at Dragon Boat Festival, making mooncakes at Mid-Autumn Festival, etc.).

Voluntary services are a crucial aspect of long-term care and greatly complement the resources provided by the government,these nursing home organizations welcome volunteers who perform various non-medical activities associated with the daily lives of older adults [10–12]. Volunteers offer additional assistance and companionship to residents, provide support to employees such as nurses, nutritionists, and physical therapists, and potentially improve the overall quality of care, in China, these services have become increasingly popular and play a crucial role [13, 14]. However, some problems have emerged in voluntary services, The voluntary services provided by volunteer organizations for older adults have certain functional defects and efficiency dilemmas, such as an unsound volunteer management system, high mobility of volunteers, and lack of a corresponding volunteer training system, which leads to the inability to provide high-quality services [13]. The above problems have undermined the effectiveness of voluntary services and affected the regular operation of nursing home organizations [15].

For effective services for older adults, it’s critical to understand the needs and experiences of older adults in nursing home organizations, there have been limited studies on how older adults feel about receiving voluntary services and if such services are suitable for their actual needs. One qualitative study documented the experiences of older adults who were helped by volunteers, but it was mainly focused on the volunteers themselves [16]. Another study looked into the benefits and experiences of receiving voluntary services, but it specifically focused on older adults who were confined to their homes [17]. There is no research available that sheds light on the emotions and requirements of older adults who receive voluntary services in nursing home organizations. To bridge this gap, we conducted interviews with older adults who have been accepted for voluntary services in two nursing home organizations in Hangzhou. The objective of this study is to gain a deeper understanding of the actual needs and experiences of older adults and use this information to guide promoting the effective growth of voluntary services and establishing a voluntary service system that is suitable for older adults in nursing home organizations.

## Methods

### Design

This study adopts a qualitative descriptive approach to examine the experiences and expectations of older adults in nursing home organizations when receiving voluntary services. This study aims to gain a comprehensive understanding of the actual experiences and needs of older adults residing in nursing home organizations regarding receiving voluntary services and explore the types of voluntary services that are most suitable for the needs of older adults. To ensure accuracy and transparency, the authors followed the Consolidated Standards for Reporting Qualitative Research (COREQ) guidelines when reporting their findings [18].

### Participants

During June-August 2023, the authors used purposive sampling to sample older adults residing in two nursing home organizations in Hangzhou, the inclusion criteria for the interview subjects were as follows:

1. they had to have resided in the nursing home organizations for more than a year;
2. they had to have received voluntary services;
3. they had to be conscious and able to express themselves effectively;
4. they had to have given informed consent and voluntarily agreed to participate in the study.

The number of people participating in the study was decided based on information saturation, this means the interviews were conducted until no new topics emerged and responses were repeated, the data from the twelfth interview indicated that saturation had been reached as confirmed by the other two interviews. This research principle was based on previous qualitative research studies [19]. A total of 14 older adults, coded N1-N14, were included in this study. All older adults who participated in the study agreed to the interview process, and none withdrew during the study. Detailed information can be found in Table 1.

**Table 1:** General information of the interviewees (*N* = 14)

Number	Gender	Age (years)	Educational level	Occupation	Marital status	Length of residence (years)
N1	Female	78	Junior high school	Accountant	Widowed	6
N2	Female	81	Secondary school	Doctor	Married	3
N3	Female	80	Junior high school	Worker	Married	2
N4	Female	75	Junior high school	Teacher	Married	1
N5	Female	90	College	Teacher	Widowed	3
N6	Male	83	College	Teacher	Widowed	2
N7	Male	79	Junior high school	Worker	Married	4
N8	Female	75	Junior high school	Teacher	Married	3
N9	Male	77	Junior high school	Worker	Widowed	5
N10	Female	88	Secondary school	Veteran	Widowed	3
N11	Male	76	College	Engineer	Married	2
N12	Male	89	University	Cadre	Widowed	2.5
N13	Male	83	Junior high school	Worker	Widowed	4
N14	Female	81	Junior high school	Worker	Widowed	2

### Interview outline

We developed an interview outline after thoroughly reviewing the literature sources and consulting with the research group [20, 21]. We selected two older adults living in nursing home organizations to conduct pre-interviews, we adjusted the interview outline based on the feedback we received from the pre-interviews.

The interview will cover the following topics:

1. Please describe the voluntary services you have received in detail. How do you feel about receiving these services?
2. Are you satisfied with the voluntary service you have received? What aspects of the service make you satisfied?
3. What are your dissatisfactions with the voluntary service? Why do you feel that way?
4. What are your expectations and needs for the voluntary service’s content, form, and volunteers?
5. Is there anything else you would like to add to the discussion?

### Data collection

A semi-structured interview method was utilized to gather data for this study. The main researcher, (a master’s degree nursing student) has been trained in qualitative research methods and has mastered the semi-structured interview techniques required to conduct interviews independently. Additionally, the researcher has participated in various volunteer activities in nursing organizations and has established a trustworthy relationship with the interviewees. Before conducting the interviews, the main researcher explained the study’s purpose and methodology to the interviewees and, after acquiring their consent, scheduled an appointment in advance. Face-to-face interviews were conducted with the respondents in a quiet, private, comfortable conference room. During the interview, the researcher recorded the entire process with the respondent’s consent without interrupting the respondent unnecessarily. The researcher confirmed the key concerns and the content that the respondent could not express clearly by repeating, asking follow-up questions, and asking rhetorical questions. The researcher also promptly recorded the respondent’s non-verbal information, such as movements, expressions, and tone of voice. Each interview lasted 30–45 min, and after conducting 14 interviews, no new information was obtained, indicating data saturation and ending the interview process. At the end of the interview, each interviewee was given a small token of appreciation.

### Data analysis

The audio recordings of the interviews were transcribed into text within 24 h of completion, non-verbal information was noted in the transcript at relevant places. The transcribed information was then entered into the NVIVO 11.0 software (QST International, Cambridge, MA, USA) for data extraction, coding, and integration. Two researchers independently analyzed and coded the data, and the results were compared to identify common themes. Any discrepancies were resolved after the research team had discussed them to ensure that the data was complete and the analysis was accurate. Colaizzi’s seven-step analysis method was used to refine the themes from the interviews, which involved the following steps [22]:

1. Carefully read all the transcriptions of the interviews.
2. Analyze the significant statements made by the interviewees.
3. Code the recurring and meaningful ideas discussed in the interviews.
4. Gather the coded ideas and form the theme clusters.
5. Define and describe the themes from the coded ideas.
6. Identify similar ideas and sublimate the theme concepts.
7. Return the results to the interviewees for verification, and revision, and add the results based on the feedback from the interviewees. For detailed coding results, please see Table 2.

**Table 2:** illustrates the themes, categories within themes, codes, and examples

Theme	Category	Code	Examples
Experiences	Beneficial experiences	Solving problems and meeting needs	*N11: “When the volunteers come to teach me how to use computers, I ask them something I don’t understand, and the teacher will explain it to me immediately.*
		Feel warmth and care	*N10: “I am delighted when I participate in volunteering, I feel that I have a group life again;,I am pleased, I feel that someone cares about us.”*
	Unpleasant service experiences	A formality that makes it difficult to benefit truly	*N5: “Some volunteers are very perfunctory, they come for a while and leave quickly.”*
		Lack of organization, regularity, sustainability	*N5: “Volunteers can’t come regularly, they come once in a while or not regularly and don’t have a plan.”*
		The mismatch between service provision and actual demand	*N10: “Some volunteers come just to dance and sing; it feels very noisy, I don’t want to participate, I want the volunteers to talk to me peacefully and quietly.”*
Needs for volunteerism	Needs for emotional comfort	The desire for companionship and care	*N1: “I hope someone will come and chat with us.”*
		The need for psychosocial support	*N1: “Many older adults have no way to contact the outside world, so they have psychological barriers, they need psychological counseling. ”*
		Desire to increase contact with the community	*N12: “I would like volunteers to communicate with us, tell us what is happening outside, tell us something new.”*
	Cultural and recreational needs	Cultural and recreational teaching needs	*N14: “It’s good for volunteers to come and teach older adults how to dance, sing, and sing opera, and time passes a little faster when we all get together and learn.”*
		The need for viewing cultural performances	*N2: “It is popular for volunteers to bring cultural performances to our nursing home, we love to see young people performing programs, singing some classic old songs or Peking Opera, it is very popular.”*
		Physical fitness needs	*N9: “We would like to play tai chi, it is a very suitable sport for us as it strengthens the body and the movements are softer, it would be nice if a teacher could teach us.”*
	Knowledge acquisition needs	Use of electronic equipment	*N1: “It’s become very convenient to buy things online, but I don’t know how to operate it myself and would like someone to teach me.”*
		Healthcare knowledge learning	*N9: “Volunteers can come and talk to us about medicine and how to predict dementia.”*
		Laws and regulations on older adults	*N12: “Volunteers can help us learn how to write a will effectively and can avoid unnecessary trouble and conflicts in the future.”*

## Results

After the data analysis was summarized, two main themes were identified: Experiences and Needs for volunteerism.

### Theme 1: experiences

#### Beneficial experiences

##### Solving problems and meeting needs

Many older adults currently reside in nursing home organizations that are situated far away from their children and friends, they often face difficulties in getting help promptly when they encounter problems, which can affect their daily lives. For instance, in today’s rapidly developing society, many older adults own smartphones but lack the necessary knowledge to use them effectively. This, in turn, reduces their social participation and increases their sense of isolation. However, voluntary services have been instrumental in assisting them in overcoming these hurdles and leading a more fulfilling life.

*N11: “When the volunteers come to teach me how to use computers, I ask them something that I don’t understand, and the teacher will explain it to me immediately.”*

*N1: “I don’t know how to buy things online. Volunteers taught me little by little, and after a few teaching sessions, I learned how to do it so I don’t have to bother the caregiver every time. I can also do online shopping by myself, and I feel that life is much more convenient.”*

Some respondents stated that volunteers could fulfill their needs. Professional volunteers also taught older adults Chinese medicine and health care and assisted with self-care.

*N12: “I’m interested in Chinese medicine health care knowledge, and when students from the University of Traditional Chinese Medicine come over, and I ask them What are the functions of different acupoints, they tell me how to press them to make them work.”*

##### Feel warmth and care

Many older adults live in nursing home organizations, away from their familiar environment and social network. This isolation can generate a sense of loneliness, making them more eager for emotional support. Volunteers provide services to add joy to the lives of older adults so that they feel cared for. Interviewees have mentioned that being taken care of on their initiative makes them feel warm and touched, increasing their overall sense of well-being.

*N10: “I am delighted when I participate in volunteering, I feel that I have a group life again, I am pleased, I feel that someone cares about us.”*

*N8: “Volunteers come to serve us, feel that people still care about us older adults, and now the country also cares about us, and society also cares about us, I am thrilled.”*

Some respondents said that having someone to talk to and greet them would make them feel happy and that they were willing to communicate with young people and accept their new ideas.

*N2: “As soon as I see you young people, I am happy, I feel the atmosphere of youth, my mood is different, I feel less lonely.”*

#### Unpleasant service experiences

##### A formality that makes it difficult to benefit truly

According to the interviewees, there are certain formalized phenomena in the domain of volunteering. Some volunteers engage in volunteering activities to obtain a certificate, such certificates can help them get extra points at work. Some volunteers participated in volunteering based on the mentality of the herd under the organizational arrangements of their schools or enterprises. These volunteers lack initiative, violate the principle of voluntarism, and cannot provide services that genuinely benefit older adults due to their single-mindedness and formalism during the service process. As a result, older adults have a poorer sense of experience.

*N7: “Some volunteers are asked to serve by their companies, and they have to finish the job; some just go through a process.”*

*N13: “Many volunteers come over to perform a show, then take photos and leave; the service time is very short, just like completing a task.”*

*N5: “Some volunteers are very perfunctory; they come for a while and leave quickly.”*

##### Lack of organization, regularity, sustainability

Many volunteers offer their services without compensation, while they have their formal jobs, which makes it difficult for them to provide services consistently. Additionally, volunteers may be more mobile, which can result in a lack of continuity in the services that are provided and the target groups that are served. However, older adults living in nursing home organizations often have monotonous and lonely lives, and occasional voluntary services may not be enough to meet their needs. As a result, some older adults may feel dissatisfied with the irregular and unsustainable nature of voluntary services.

*N12: “Volunteers come on an ad hoc basis; they are not regular. Recently, a school teacher came to teach us how to sing, but unfortunately, they had to leave due to commitments and have not been able to come back.’’*

*N5: “Volunteers can’t come regularly; they come once in a while or not regularly and don’t have a plan.”*

*N7: “Volunteers come to the nursing home occasionally, so they don’t want to bother them.”*

##### The mismatch between service provision and actual demand

The voluntary services provided to older adults in nursing institutions were not able to match their real needs as the volunteers had no prior knowledge of their needs and did not make any advance preparations.

*N4: “Last time, a volunteer came and asked me if I needed help with cleaning. However, I declined their kind offer because caregivers in the nursing home clean rooms every day, and the volunteers could not address the specific things I needed help with.”*

The needs of older adults for volunteering can vary significantly based on their experiential backgrounds, and physiological and psychological conditions. Therefore, providing the same services to all older adults can lead to negative feelings towards volunteering among them.

*N10: “Some volunteers come just to dance and sing, it feels very noisy. I don’t want to participate, I want the volunteers to talk to me peacefully and quietly.”*

*N14: “I am not very good with my legs, so it is difficult for me to participate in activities organized by the volunteers downstairs. I would like to find activities I can participate in in my room, such as playing games or doing crafts.”*

### Theme 2 needs for volunteerism

#### Needs for emotional comfort

Many older adults live in semi-closed institutions where they lack long-term support from their families and struggle to find someone to talk to. During the epidemic, nursing home organizations prohibited visitors to prevent the spread of the virus, leaving many seniors alone and cut off from the outside world. As a result, many older adults experience feelings of loneliness and depression. To help combat these negative emotions, volunteers can provide companionship and support, which can effectively reduce feelings of loneliness and promote emotional well-being.

*N1: “I hope someone will come and chat with us; many older adults have no way to contact the outside world, so they have psychological barriers, they need psychological counseling, they need someone to come and chat with them to relieve their loneliness.”*

*N10: “It’s better to have volunteers to come over to the service, to come and chat with me, to visit me.”*

*N12: “I would like volunteers to communicate with us, tell us what is happening outside, tell us something new.”*

#### Cultural and recreational needs

As people age, their social interactions tend to decrease, and they gradually tend to withdraw from daily life. This results in older adults having more free time after their retirement. Nursing home organizations can provide basic living care and medical assistance for older adults, which relieves them of the burden of cooking, cleaning, and shopping. This also means they have more free time than those who live at home or in the community. Many older adults wish to participate in cultural and recreational activities, such as singing, dancing, sports, and watching performances, to add excitement to their lives. They hope that volunteers can organize such activities to help them reduce their loneliness and spend their time in a meaningful way.

*N14: “It’s good for volunteers to come and teach us how to dance, sing, and sing opera, and time passes a little faster when we all get together and learn.”*

*N2: “It is popular for volunteers to bring cultural performances to our nursing home, we love to see young people performing programs, singing some classic old songs or Peking Opera, it is very popular.”*

*N9: “We would like to play tai chi, it is a very suitable sport for us as it strengthens the body and the movements are softer, it would be nice if a teacher could teach us.”*

#### Knowledge acquisition needs

According to Maslow’s Hierarchy of Needs Theory, individuals will naturally shift their focus toward higher-level pursuits once their basic and low-level needs are met. In the case of older adults residing in nursing home organizations, their basic material needs are taken care of, and as a result, their need for knowledge and learning becomes increasingly important. Many older adults require assistance in learning how to use electronic equipment, which can help facilitate their communication with the outside world and reduce feelings of isolation.

*N1: “It’s become very convenient to buy things online, but I don’t know how to operate it myself and would like someone to teach me.”*

*N2: “My daughter bought me an expensive Apple phone, but I am unfamiliar with how to use it. It would be great if someone could systematically instruct me on how to use the smartphone.”*

*N8: “I don’t know how to use my smartphone, I don’t understand many functions, so I would benefit from having a teacher to guide me.”*

As individuals age, their bodily and cognitive functions may deteriorate, adversely affecting their quality of life. Basic healthcare knowledge can be critical for older adults to maintain good health. Many older adults have a strong desire to learn about nutritional diets, rational exercise, and traditional Chinese medicine physiotherapy as a means of improving their health.

*N9: “Volunteers can come and talk to us about medicine and how to predict dementia.”*

*N13: “I have high blood pressure and cholesterol. I need advice on what to eat and what to avoid.”*

To prevent any disagreements regarding the distribution of their assets among their heirs after they pass away, older adults seek the help of volunteers to assist them in drafting a will that is by national policies and regulations and has legal validity.

*N12: “Volunteers can help us learn how to write a will effectively and can avoid unnecessary trouble and conflicts in the future.”*

## Discussion

### The current situation of voluntary experiences of older adults in nursing home organizations

#### Analysis of beneficial experiences

The study’s findings indicate that individuals residing in nursing home organizations who are of advanced age have mixed experiences when it comes to receiving voluntary services. Most respondents conveyed the warmth and care emanating from the volunteers and the society towards older adults. Furthermore, they shared that volunteering offered them a means to engage in activities actively, create connections with fellow older adults, and foster mutual support and camaraderie. This social participation has the potential to enhance the mental well-being of older adults, thereby decreasing feelings of loneliness and depression [17]. Voluntary activities like smartphone training can help older adults acquire the necessary needed skills and adapt better to modern technology and life. Competent skills are crucial for older adults, particularly in today’s fast-developing technological society, where electronic devices such as smartphones are becoming increasingly popular. However, many older adults need more skills to operate these devices and thus cannot fully utilize them. Through training, older adults can learn how to use smartphones, including sending text messages, browsing the web, using social media, downloading applications, and more. Learning these skills not only improves the quality of life of older adults but also helps them stay connected with family and friends, thereby reducing loneliness.

Improved skills can assist older adults in accessing and utilizing health information, including online medical advice and health apps. This information can aid in managing their health status, preventing and managing chronic illnesses, and ultimately improving their quality of life. Volunteering is crucial in nursing home organizations. It provides numerous benefits to older adults, including enhancing their mental health and quality of life and receiving the necessary support and care by participating in voluntary activities [23].

#### Analysis of unpleasant experiences

During the interviews, some older adults shared negative experiences regarding the content, form, and frequency of voluntary services. They pointed out that volunteers did not understand their needs in advance, focusing too much on material assistance and neglecting their psychological and intellectual needs. Additionally, the service process is often too process-oriented and formalized, with less interaction with older adults, resulting in voluntary services failing to meet their expectations.

Research suggests that negative experiences of receiving voluntary services may impact older adults’ willingness to seek help and the effectiveness of voluntary services. Therefore, when providing voluntary services to older adults, it is essential to take the initiative to understand their experiences and continuously optimize the voluntary program. This approach is crucial to improving the quality of voluntary services [24].

### The current situation of the demands for voluntary services by older adults

The study results show that nursing home organizations can provide comprehensive life care services to older adults, meaning they do not require many voluntary services for life care. However, this does not imply that older adults’ needs are met. Their need for emotional support, cultural recreation, and knowledge-seeking and learning is highly concentrated.

When older adults leave their familiar family environment to move into care institutions, they may experience feelings of loneliness and boredom due to the lack of regular interaction with their children, family members, and friends. This sense of isolation can harm their mental health, and they may seek more opportunities to communicate and interact with younger individuals to gain emotional comfort [25].

As people age, cultural entertainment and knowledge learning become essential for spiritual growth. After their basic living needs are taken care of, older adults desire more fulfilling recreational activities, such as calligraphy, painting, and singing, these activities enrich the spiritual life of older adults and benefit their physical and mental health [26].

In today’s rapidly developing society, the widespread use of smartphones and the popularity of online shopping have led to a digital divide among older adults. This phenomenon has, to some extent, hindered their social participation and increased their sense of isolation. Consequently, there is a growing demand for voluntary services that assist with smartphone use and can help them enjoy a convenient and fulfilling digital life.

The need for voluntary services for older adults has changed over time. While they still require help with their daily living, they also need emotional support, cultural engagement, and opportunities to learn new things. We should focus on meeting these needs to ensure our voluntary services are beneficial. By doing so, we can help older adults live fulfilling, healthy, and happy lives in their later years [27].

### Suggestions and strategies for optimizing volunteerism

#### Accurately assessing older adults’ voluntary service needs

The study results reveal that some older adults have negative experiences with voluntary services that fail to meet their actual needs, leading to unsatisfactory service outcomes. This highlights the need to accurately identify the real service needs of older adults to improve the quality and effectiveness of voluntary services.

To achieve our goal, we need to take a series of steps. Firstly, we must create appropriate tools for evaluating the needs of older adults for voluntary services. We should also clarify the assessment methods and strategies for assessing these needs, before launching voluntary services, relevant organizations and volunteers must understand older adults’ service experience and needs through qualitative and quantitative assessment methods [28].

To improve the quality and effectiveness of voluntary services for older adults, we can utilize big data technology to carry out precise reforms. This involves building a unified information platform for voluntary services that enables a quick match between the needs of older adults and the specialties of volunteers through the co-construction, sharing, and everyday use of resource information [29]. By doing so, we can provide multi-level, multi-category, and personalized voluntary services that cater to the actual needs of older adults, thus achieving the purpose of “precise service.”

In conclusion, we must prioritize the actual needs of older adults and provide them with more personalized and intimate voluntary services by continuously improving the assessment tools and information platforms with the orientation of precise services, the use of big data technology will play a key role in helping us realize the goal of efficient and accurate services.

#### Improving the quality management system of voluntary services

Volunteering quality refers to the quality of services volunteers provide, as perceived by the direct recipients. Research has shown that low-quality voluntary services fail to achieve their intended goals, moreover, negative experiences of receiving voluntary services may discourage older adults from seeking help in the future. The study highlights a significant gap between older adults’ experience of volunteering quality and their expectations, therefore, it is necessary to strengthen the management of volunteering quality to ensure that expectations are met.

To enhance the quality of volunteering, we need to implement measures. Firstly, we must optimize the recruitment and selection system for volunteers, this entails formulating recruitment plans and selection requirements that align with the voluntary services needs of older adults. We aim to create a stable and committed volunteer team skilled in services knowledge and job skills and willing to participate in voluntary services for an extended period [30].

To enhance the level and quality of service, it is important to provide regular and standardized training to volunteers. Volunteers should receive professional information support services, such as training on volunteer spirit, etiquette, communication skills, and the physiological and psychological characteristics of older adults. The main forms of training include information consultation, professional knowledge, technology lectures, sharing of previous volunteer experiences, summarizing stage-by-stage voluntary services, and experiential services. Volunteers should be provided with face-to-face or online interaction to help them improve their ability to assist older adults. The training for volunteering encompasses theoretical knowledge about volunteering, including its characteristics and principles, the rights and interests of service users, and respect for them. It also includes basic knowledge of social work, such as interpersonal communication methods and skills, as well as knowledge of health care for older adults. The latter includes the introduction of general knowledge about daily life care for older adults, such as diet, hygiene, and exercise, and the evaluation of the training’s effectiveness. Both voluntary service organizations and nursing home organizations should participate in the training process, only volunteers who have completed the training and assessment can engage in service activities [31]. It is essential to improve the evaluation mechanism of voluntary service quality. This can be done by creating a scientific evaluation index system involving older adults in evaluating their satisfaction with the voluntary service program and conducting a comprehensive analysis of the evaluation results. This analysis can help to optimize and improve the service program, additionally, tracking and evaluating the effectiveness of optimization measures to continuously enhance service quality is crucial [32].

Improving the quality of voluntary services is a comprehensive project that enhances various aspects, such as volunteer recruitment, training, and service quality evaluation. This systematic approach can help serve the nursing home organizations better and improve their overall quality of life.

### Strengths and limitations

The paper’s strength lies in its focus on the experience of older adults in nursing institutions when receiving voluntary services and their need for such services. This study’s understanding of the real feelings and needs of older adults is beneficial for various organizations in society to provide better services in a targeted manner. However, the study’s limitation is that it mainly focuses on the more developed areas of Hangzhou, which affects the sample’s representativeness and makes it challenging to reflect the general situation of older adults in nursing home organizations. Additionally, the author’s subjective viewpoints may affect the analysis of the material during the data analysis process. Finally, the sample size of this study is relatively small, and there may be individual differences in personality, physical condition, and economic situation, among others. Therefore, expanding the sample size and the region’s scope to carry out more in-depth research is necessary.

## Conclusion

This research explored the experiences and requirements of older adults who receive voluntary services in Chinese care homes. The study categorized their experiences into two groups: beneficial experiences and unpleasant service experiences, the needs of older adults who receive voluntary services include emotional comfort, cultural and recreational, and knowledge acquisition. It is crucial to have a timely and comprehensive understanding of the experiences and needs of older adults to create a targeted voluntary service model, standardized management, and training of volunteers in nursing home organizations.

### Electronic supplementary material

Below is the link to the electronic supplementary material.

Supplementary Material 1

Supplementary Material 2

## Data Availability

The datasets used and/or analyzed during the current study are available from the corresponding author upon reasonable request. The datasets are not publicly available due to confidentiality and ethical restrictions.
