# Supplementary figures and images for: The experiences and needs of older adults receiving voluntary services in Chinese nursing home organizations: a qualitative study
Source: BMC Health Serv Res. 2024 Apr 29;24:547. doi: 10.1186/s12913-024-11045-5 (PMC11059588; doi:10.1186/s12913-024-11045-5)

This is the ethical review of this study


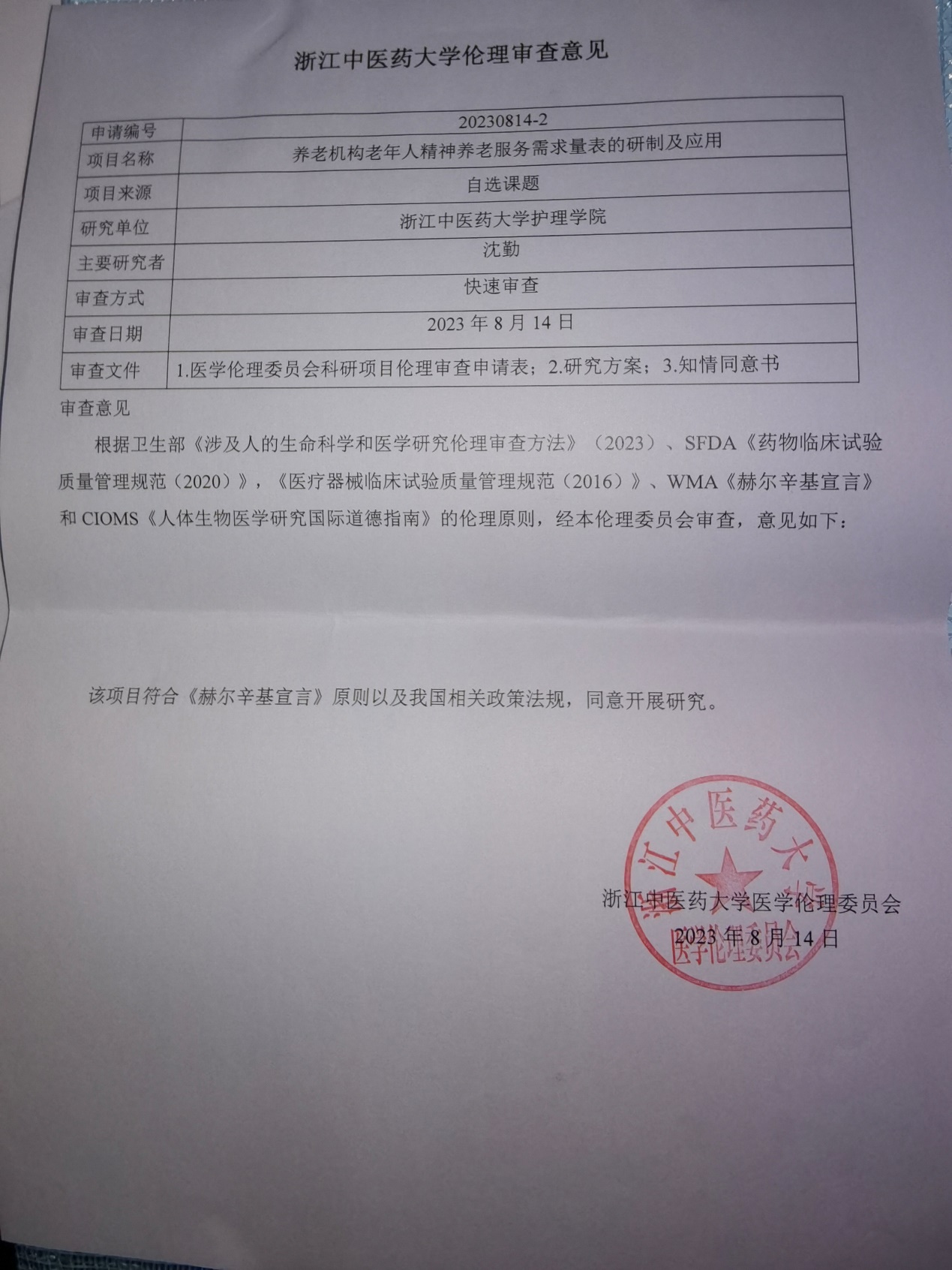

Supplement: Supplementary file 1 — Supplementary Material 1 [file 12913_2024_11045_MOESM1_ESM.docx]
