# Supplementary material for: The experiences and needs of older adults receiving voluntary services in Chinese nursing home organizations: a qualitative study
Source: BMC Health Serv Res. 2024 Apr 29;24:547. doi: 10.1186/s12913-024-11045-5 (PMC11059588; doi:10.1186/s12913-024-11045-5)
Supplement: Supplementary file 2 — Supplementary Material 2 [file 12913_2024_11045_MOESM2_ESM.docx]

outline of the interview

1. Please describe the volunteer services you have received in detail. How do you feel about receiving these services?

2. Are you satisfied with the volunteer service you have received? What aspects of the service make you satisfied?

3. What are your dissatisfactions with the volunteer service? Why do you feel that way?

4. What are your expectations and needs for the volunteer service's content, form, and volunteers?

5. Is there anything else you would like to add to the discussion?
